# The characteristics of compassionate care during childbirth according to midwives: a qualitative descriptive inquiry

**DOI:** 10.1186/s12884-020-03001-y

**Published:** 2020-05-19

**Authors:** Samantha Salome Krausé, Catharina Susanna Minnie, Siedine Knobloch Coetzee

**Affiliations:** NuMIQ Research Focus Area, North-West University, Private Bag x6001, Potchefstroom, 2520 South Africa

**Keywords:** Compassion, Care, Empathy, Kindness, Labour, Childbirth, Midwifery, Perception

## Abstract

**Background:**

Although compassion is considered to be of prime importance in nursing and midwifery, there is no clear understanding of what compassionate care in childbirth entails, and how midwives perceive compassionate care is largely unknown. This study accordingly seeks to describe the characteristics, of compassionate care during childbirth as perceived by midwives.

**Methods:**

A qualitative descriptive inquiry was undertaken with a voluntary online survey, where participants were recruited via snowball sampling on the social networking site, Facebook. The participants were midwives, and the unit of analysis was the received responses. Participants reported on instances of compassionate care during childbirth. The data was thematically analysed using Tesch’s eight steps to identify common themes.

**Results:**

Ninety-eight responses were analysed and three themes with eight sub-themes emerged as dominant characteristics. Themes and sub-themes were as follows: making meaningful connections with women (displaying good interpersonal skills, conduct based on dignity and respect, establishing trust); initiating individualised understanding of each woman (showing empathy, permitting maternal choice) and action through care and support (providing emotional support, assistance through instrumental care, continuous informational support).

**Conclusions:**

In seeking to determine what characterises compassionate care in childbirth, the findings reveal a process of making meaningful connections with women through recognising their needs, initiating individualised understanding of each woman’s needs and desiring to ease it, which is subsequently manifested in action through emotional, instrumental and informational care and support. A better understanding of how midwives perceive compassionate care could potentially improve the quality of care midwives offer during childbirth.

## Introduction

Midwives work together with women during their pregnancy, labour and puerperium stages, and global standards have been set for the minimum cognitive and psychomotor competencies required of a midwife [1]. In the midwifery literature, a recent shift has increased attention to affective competencies, with particular focus on the notion of the “good midwife”. A “good midwife” is a term applied to a midwife who is competent with the cognitive, psychomotor and affective domains. Competency in the affective domain has been described as emotional intelligence, a caring personality and the use of emotions and feelings such as compassion, kindness and empathy [2–4] of which compassion is considered of prime importance [5] and pivotal to quality nursing and midwifery care [6, 7]. Although researchers are exploring the term compassion in nursing [5, 6], little is known about what constitutes compassionate care in midwifery [7], which is essential to improve midwifery care [2, 4, 7] and decrease the incidence of disrespect and abuse [8].

## Background

Midwives are often the main caregivers for healthy women during childbirth [9–11] and have been referred to as the “backbone” of maternal and newborn care [9]. Globally, the International Confederation of Midwives (ICM) guides midwifery associations and their Governments on the minimum cognitive and psychomotor competencies required of a midwife [1, 2, 4, 11]. Once deemed competent, the midwife may practice in whichever context is applicable: the home; the community; hospitals; clinics or health units [1]. The competent midwife then works together with women, taking full responsibility and liability for their pregnancy, labour and puerperium care [11].

How the midwife cares for the woman during childbirth is associated with deep personal and cultural significance for the woman herself and for her family [12]. Such care, requires competency in the affective domain and has been described as emotional intelligence, a caring personality and the use of soft skills, such as compassion, kindness and empathy [2–4]. Compassion is considered to be so important in the affective competencies of a registered midwife that suggestions have been made to add it as a core competency in midwifery care [13–15]. A “good midwife” so termed, is one who is competent in the cognitive, psychomotor and affective domains [2, 4, 16].

Recently, there have been an alarming number of reports of disrespect and abuse during childbirth [8, 12, 17–21]. This has been noted by the World Health Organization (WHO) as a serious problem that needs urgent attention [22]. Every woman has the right to the highest attainable standard of health, which includes the right to dignified, respectful care [23]. A recent WHO advisory on positive childbirth experience makes recommendations on how to manage and minimise disrespect and abuse by providing compassionate care to all women [24].

Compassion is defined as feeling together [25], feeling with another [26], and subsequently taking action or performing deeds of kindness [27] to provide relief for suffering or pain [7, 26, 28]. It has been cited as possibly the most highly valued attribute in nursing and midwifery care [6, 26, 29] and is widely recognised as its core element [25, 26, 30–33]. Compassion is described as a constituent of nursing excellence [29] and as a vital component for achieving quality nursing and midwifery care [5, 14, 34–37].

Compassion is thus undeniably sought after in nursing and midwifery care [29, 31]. Yet how compassion manifests itself in provision of care is poorly understood, as it differs from practitioner to practitioner and from practice setting to practice setting [5, 26, 32, 36, 38]. Although researchers are exploring the term compassion in nursing [5, 6, 28, 31, 34, 39];, little is known about what constitutes compassion in midwifery [7]. This study was accordingly undertaken to describe midwives’ perceptions of compassion in caregiving for women in childbirth.

## Design

A qualitative descriptive inquiry is a label used in qualitative research for studies which are explorative and descriptive in nature, particularly for examining health care-related phenomena, that are focused on discovering the who, what, where, when and how of events or experiences and gaining insights from participants regarding poorly understood phenomenon [40, 41]. According to Sandelowski [2000], “Qualitative descriptive study is the method of choice when straight descriptions of phenomena are desired.” A qualitative descriptive inquiry was chosen in response to a deficit in the understanding of the concept of compassionate care in midwifery.

## Methods

The research methods for this study as briefly outlined below include population and sample, data collection, data analysis, ethical considerations and trustworthiness.

### Population and sample

The target population was midwives. They were recruited using a snowball sampling technique, via Facebook groups and pages. Facebook was chosen as the social networking site best suited for recruitment of potential participants in view of its size, features, intensive use, and continuing growth [42]. Social networking sites offer new ways for researchers to conduct studies quickly and cost-effectively, particularly in constructing snowball samples for exploratory and descriptive work [42–44].

First the researchers created a Facebook page entitled, “Promoting kind and compassionate care during childbirth.” The Facebook page contained all the information about the research project and a direct link to the SurveyMonkey electronic platform that was used to host the self-administered survey. To begin the recruitment process, the researchers searched Facebook for all midwife-based groups and pages using the search words “midwife” and “childbirth” (*N* = 86). The researcher then posted a notice on every midwifery group and page, informing them about the study and its purpose, and inviting them to participate in the study via a link to the study’s Facebook page. Typically, each Facebook user has their own network of friends, family and Facebook groups (virtual communities linking people with common interests). Midwives of the selected groups and pages, were further encouraged to like and share the study’s Facebook page; this increased the likelihood of other midwives taking cognizance of the study, thereby propelling the snowball sampling process.

The units of analysis were the responses from each midwife, not the midwives themselves. Inclusion criteria for midwives were experience in assisting women during childbirth and having internet access (more specifically, a Facebook account). As the aim of the study was to gain understanding about compassionate care in midwifery, no limit was placed on the number of responses received. If there were more than one incident midwives wished to report, they were invited to do so, since any incident profound enough to make an impact on the midwife was deemed relevant. For this same reason, there were no exclusion criteria.

The researcher could not predict a specific sample size due to the descriptive nature of the research problem and the unpredictability of the number of replies. Hill [1998] indicates a wide range of possibilities (from 30 to 500) for recommended sample size in an online survey, with a small sample size being more economical while a larger sample adds reliability and representativeness [45]. This study had 98 completed responses, which was considered to be sufficient, as data saturation took place.

The survey was open for 3 months for data collection from the 13th of December 2016 to the 13th of March 2017. In an online survey reported by Bhutta [2012], the majority of the responses were received within the first few days following the survey launch [46]. However, in this study, 50 responses were received within the first month. A second invitation was therefore posted on the Facebook page, and a further 48 responses were then received over the subsequent 2 months.

### Data collection

Data were collected through a voluntary, anonymous online survey. Electronic surveys have been reported as equivalent to paper-based surveys in terms of internal reliability and completion rates [46, 47]. The benefits of online surveys are that they save time, are cost-effective, and can be accessed quickly by people with shared characteristics from widely separated geographical locations [48, 49]. SurveyMonkey was chosen as the online site to collect and store data in view of favourable features it offers such as affordability, easy format and possibility to set up the survey as desired [50].

The self-administered survey consisted of a demographic section with five closed-ended questions and three open-ended questions. According to Callegaro et al. [2015], an advantage of open-ended questions is that participants have an opportunity to re-tell a life event that made an impact on them in as much detail as needed, thereby revealing how the concept is perceived (in this case, by the midwife). Closed-ended questions, on the other hand, do not provide much insight. The disadvantage of open-ended questions is that it might be a cognitive strain to answer them; they were accordingly kept to a minimum in this study [44]. The survey was not time-consuming, as the expected completion time was 15 to 30 min.

The closed-ended questions (with the options for choice of one standard answer indicated in brackets) were as follows:

- What is your gender? (male or female)
- Where do you practice midwifery? (rural area or urban area)
- In which facility do you practice midwifery? (state hospital, private hospital, community health centre, private practice that is midwife-lead or other)
- How many years’ experience do you have working as a midwife? (1–5 years, 5–10 years, 10–20 years, 20–30 years or 30 plus years)
- Is this the first time you are answering the survey? (yes or no).

Concise instructions were given, followed by the three open-ended questions. Two were explicitly phrased:

- Please write a paragraph about an instance in which you saw or experienced kind and compassionate care during childbirth.
- List five words that you associate with kind and compassionate maternity care.

The third made provision for participants to contribute any additional comments. These questions were pilot tested with student midwives. Once the survey was complete, the participant was asked to click on the “submit” button.

### Data analysis

Thematic analysis was carried out according to Tesch’s eight steps as explained by Creswell [2009]. Thematic analysis was the most appropriate means to analyse this data set, as the objective of the study was to describe the characteristics of a specific concept [40]. Braun and Clarke [2006] explain that thematic analysis is “a method of identifying, analysing and reporting patterns within data.” Patterns can also be referred to as themes [51]. This makes it possible to identify a common train of thought in the responses received from the participants [52].

The data analysis took place as follows: the data was transferred to an Excel spreadsheet, thereafter imported into the qualitative research programme Atlas.ti 8. The programme was used to process the large amount of data into a smaller set of easily readable interpreted themes [53]. The data were managed and arranged by numbers according to the sequence in which responses were received. As this was an anonymous survey, no other identifying criteria were available.

Tesch’s eight steps as explained by Creswell [2009] were carried out as follows in the thematic analysis: the data set was read carefully to get an overall sense of the data. Next, it was looked at in greater detail to begin the coding process. Coding is defined by Creswell [2009] as the process of taking a large amount of data and breaking it up into smaller sections before attaching meaning to it. These smaller sections were named following the open coding process of giving labels or renaming sections of the data set to find and categorise shared meanings. The most interesting responses from participants were identified and explored to determine their fundamental meaning. A list of topics surfaced that had been brought up several times by different participants. New categories originated from reading through the responses from the participants again. These were then refined and grouped according to similar meanings, resulting in a list of final categories – labelled as themes– which had come to light [40].

### Ethical considerations

Ethical approval was granted by the Health Research Ethics Committee of the North-West University (NWU-00072-16-A1). As the study took place online, no further legal authorization or goodwill consent was necessary.

The link to SurveyMonkey took potential participants to an introduction page which was simultaneously utilized as electronic informed consent. On this page, the potential participants were formally invited to participate in the research project, and were informed of the research purpose, the inclusion criteria, the risks and benefits of the study, how anonymity and confidentiality would be safeguarded, the voluntary nature of the study and the ability to withdraw from the study at any point in time by exiting the site. The open-ended questions were also stated on the introduction page to prepare the participants of what would be expected of them in the study. Participants were asked to confirm that they were 18 years or older; understood and accepted the outlined information, and wanted to complete the survey, by clicking on the accept button. Electronic written informed consent was obtained from all participants.

Anonymity was safeguarded as no names or contact details were requested. The completed surveys were identified in the sequence that the participants submitted. The estimated level of risk for the participants was estimated to be minimal, as the focus of the study was on positive experiences. However, participants were encouraged to contact the researchers, in the event that they experienced any type of psychological distress, and then the researchers would assist the participant with a relevant referral.

### Trustworthiness

The four fundamental epistemological standards listed by Lincoln and Guba [1985] were applied to ensure the trustworthiness of this qualitative study [54]. Theoretical validity was provided by conceptualisation of the central concepts described in the study [55].

Prolonged engagement in the field of midwifery and in the collection and analysis of data, peer examination of all decisions made in the study by the study leaders, and the researchers’ authority in the field of midwifery and compassionate care enhanced the credibility of the study findings. Transferability was strengthened by providing a thorough account of the research process applied in the study and by analysing all data entries even after data saturation was reached. The dependability of the study was enhanced through a dense description of the methodology, and review, approval and monitoring from the applicable scientific committees and study leaders associated with this study. A co-coder was assigned to thematically analyse the data set. The codes were subsequently worked on individually. The researcher and the co-coder obtained inter-coder agreement regarding the open codes [40]. The researcher declares a non-biased approach to the study and applied reflexivity, increasing the confirmability of the results.

## Results

The unit of analysis was 98 responses to the online survey, yet the accounts were provided by 88 midwives (10 participants repeated the survey). A response was considered to be completed when all 10 of the questions were answered without any questions having been skipped. Only female midwives responded to the online invitation. The majority of participants indicated practicing in urban midwife-led facilities and had on average 1 to 5 years of midwifery experience. See Table 1 for the collected demographic data of the participants.

**Table 1:** Responses for demographic data

Question	Details	Total
What is your gender?	Male	0
	Female	98
Where do you practice midwifery?	Rural area	28
	Urban area	70
In which facility do you practice midwifery?	State hospital	31
	Private hospital	17
	Community health centre	7
	Private practice (midwife-led)	24
	Other	19
How many years’ experience do you have working as a midwife?	1–5 years	33
	5–10 years	14
	10–20 years	22
	20–30 years	20
	30+ years	9
Is this the first time you are answering the survey?	Yes	88
	No	10

Table 2 sets out the reported instances of kind and compassionate care during childbirth in relation to three main themes and eight subthemes.

**Table 2:** Identified themes and subthemes in compassionate midwifery care

Themes	Subthemes
Making meaningful connections with women	Displaying good interpersonal skills
	Conduct based on dignity and respect
	Establishing trust
Initiating individualised understanding of each woman	Showing empathy
	Prioritising maternal choice
Action through care and support	Providing emotional support
	Assistance through instrumental care
	Continuous informational support

### Theme 1: MAKING MEANINGFUL CONNECTIONS WITH WOMEN

Participants felt that the midwife typically connects with the woman in labour by becoming in tune with her and creating a bond. This connection flows when the midwife and the labouring woman actively collaborate with one another through interpersonal skills, dignity and respect, and establishing trust.

### Displaying good interpersonal skills

*Interpersonal skills* is a broad term indicating capacity to communicate with others through combined verbal and non-verbal communication and active listening. Participants emphasised the importance of using positive, affirming language when communicating with women and using non-verbal cues such as facial expression, tone of voice and touch.

Participant 93:

*When the client is listened to, when her feelings are taken note of...*

Participant 73:

*… A glance a look from a woman can be interpreted by a midwife and reassurance given from a look.*

### Conduct based on dignity and respect

Participants discussed experiences where the midwife overtly displayed respect and maintained an overall framework of respect, particularly respect for bodily autonomy. This reinforced the connection between the midwife and the labouring woman, and was described as an essential part of compassionate care.

Participant 39:

*… Simple gestures of covering her body to respect her dignity; acknowledging and respecting her wishes …*

Participant 47:

*… I attended a home birth … She is a modest woman so we kept her lap covered with a towel during the birth which helped her feel more comfortable. We also did not perform any vaginal examinations for this particular client and instead we watched her contraction pattern and trusted her with what she was feeling. The birth was perfect and healthy, and all appropriate customs were performed with minimal interference and with great respect from the midwives …*

### Establishing trust

In relation to the subtheme *establishing trust*, three features stood out: establishing a partnership between the midwife and the labouring woman, being non-judgmental and creating a strong sense of safety.

Participant 64:

*Caring for a hardened criminal who did not want to engage, by being kind, gentle, inclusive and treating the couple as equals, trust was created and they engaged. They were very thankful and said they have never been treated so kindly by health professionals.*

Participant 79:

*… she had the most amazing experience because the midwife trusted in her and stood by her side without judgement...*

Participant 83:

*In a homebirth situation, where the couple had got to know their midwife through the antenatal period and were therefore able to be in a place of safety, feeling trust in their carer.*

### Theme 2: INTIATING INDIVIDUALISED UNDERSTANDING OF EVERY WOMAN

Participants described compassionate care as personal, referring to the women they are caring for in labour as “my women”. Compassion in caring for labouring woman made it seem as if the midwife was caring for her own family. Participants often made the point that each labouring woman is unique and should be seen as an individual, with their own individual needs and wishes. Participants recounted numerous instances where the essence of compassion was shown in the midwife’s empathy and respecting maternal choice.

### Showing empathy

Participants recounted numerous incidents where the midwife showed how much she cared through expressions and gestures such as crying with the labouring women in moments of great sorrow and pain.

Participant 15:

*I was working in a busy labour and delivery unit. One afternoon, (it was extremely busy that day and at the end of the shift: we were all so tired), I heard the most horrific scream from the room (unlike what we heard from our patients in this particular unit), I went in and saw the Gynaecologist examine and hurt the patient. With the patient was a midwife I had the world’s respect for! She was holding the patient and crying with her, as if she herself felt this excruciating pain …*

Participant 29:

*When the midwife left the room, I went and sat next to the mom and held her hand. I understood something that day that I’m carrying with me today still … for you it may seem like just another patient having a baby. But for this mother it was her first baby. After holding her hand and wiping her face for a while, the mom appeared calmer …*

### Prioritising maternal choice

In relation to maternal choice, participants discussed the importance of the labouring woman being able to make informed choices between alternative options, and midwives not being rushed or setting time limits during the childbirth process.

Participant 92:

*On a regular basis just providing choices to women and families and offering personalized care that is tailored to each unique birth and life circumstance.*

Participant 26:

*A birth I witnessed at our private birthing facility in which the midwife conducting the birth was patient with the mom. It took long to push baby out. She never got frustrated or upset, or used abusive language.*

In instances where maternal choice was obstructed, participants described experiences where the midwife was an advocate for the woman’s choice.

Participant 15:

The midwife very calmly and professionally told the Doctor that she did not approve of her behaviour. This will stay with me forever. –––* is such a frail, tiny, humble human being with the biggest heart: exceptional compassion for her fellow human beings and patients and the lion’s-courage to stand up against bullies. She is my hero.

* Name omitted to preserve anonymity.

Participant 50:

*A midwife standing up for a woman’s birth plan in a way which shielded the woman from the negative bullying process.*

### Theme 3: ACTION THROUGH CARE AND SUPPORT

Participants explained how compassion can be shown through emotional, instrumental and informational care and support. In emphasising the importance of giving support and encouragement there were some accounts that referred to allowing the woman to follow her instincts and lead her own birth uninterrupted, providing only what is needed and wanted. Supervision and encouragement were seen as emotional support; physical interventions to provide relief were seen as instrumental care and instruction and advice were seen as informational support.

### Providing emotional support

Participants recounted numerous experiences where the midwife used encouraging words to support the woman emotionally. They also described instances where it was the midwife’s presence that gave emotional support to the woman, such as the midwife going beyond the call of duty by staying on after the end of her shift to continue that support.

Participant 17:

*… in the end the midwife stayed with the patient until she delivered, even after the midwife was done with her shift.*

Participant 91:

*When the midwife reassuring the mother to hold on through labour stages, managing pain and giving her support, and reminding the mother that it will be worth it when she holds her baby in her hands.*

Participant 32:

*Kind and compassionate care happens when a midwife and team comes around a woman and empower her to have the best birthing experience she can ever have. I see it all the time as I am doing woman centred care, allowing women to be in charge of their birth … . I see it when women can move freely during their labour and birth, never have to be by themselves and are coached and encouraged right through. Where there is no room for fear and anxiety.*

### Assistance through instrumental care

Participants spoke of action-oriented support and care shown by midwives in assistance to relieve discomfort or pain, such as administration of analgesia or physical intervention such as a massage, judged according to the woman’s need.

Participant 27:

*Wiping the brow between contractions and gently whispering how strong she is.*

Participant 51:

*Providing the birthing woman with eggs after she had given birth and she had no food in the house.*

Participant 96:

*I was delivering someone’s baby and that mother didn’t have any clothes for the baby so as midwives we donated money and clothing for the baby.*

### Continuous informational support

Participants related stories of midwives providing care and support to the labouring woman by giving her information in the form of advice, useful instructions, helpful suggestions and guidance. There was also emphasis on the importance of giving positive feedback.

Participant 81:

*She was greeted warmly by our allocated midwife and having checked the fetal heart rate remained normal, took time to explain what was going to happen next, and gave her opportunity to ask questions.*

Participant 21:

*A midwife was explaining the death of the parent’s unborn child to them with so much love and compassion that they were feeling a lot more peaceful about letting their child go. An awesome experience I will remember for the rest of my life.*

## Discussion

This study sought to identify what midwives regarded as characterising compassionate care during childbirth. What the participants described was a process of compassionate care displayed through creation of a meaningful connection with the woman, followed by individualized understanding of each woman that led to appropriate action taken in the giving of care and support. This is similar to the process of compassion outlined by Ménage et al. [2017] in their concept analysis [7]. Broadly, the process of compassion has been described as originating in recognition of discomfort in others, accompanied by a desire to ease it [7, 15, 26, 31] which is subsequently manifested in meeting the identified need.

The participants’ descriptions of the connection created resemble what has been reported in literature [15, 38] and was seen as crucial for provision of compassionate care. When compassionate care is practiced, an authentic bond is created between the practitioner and the patient, described by Byrom and Downe [2010] as “this unseen thing” [2]. Establishing connectedness in the practitioner–patient relationship is a highly affective process [7] in which the practitioner intentionally becomes accustomed to the patient [5, 39, 56], resulting in a close and meaningful relationship [57]. Compassionate practice enables therapeutic relationships to be created through interpersonal skills [36], particularly through verbal communication such as introducing oneself [57], through non-verbal communication such as eye contact, smiling and touch [36] and through being a good listener [33, 58]. Respect has been highlighted as an important characteristic of compassion [3] and of being considerate and willing to compromise [38]. Cummings and Bennett [2012] also found that a relationship based on dignity and respect results in compassionate care, seen as the foundational principle in how to care for others [14]. Crawford et al. [2014] use the term non-judgmental to describe a characteristic of a “compassionate mentality” [59]; however, applying this term as a characteristic of kind and compassionate care during childbirth appears to be a unique finding.

In relation to midwives establishing meaningful connections, issues that participants raised included being attentive to the patient [60], focusing on the individualized understanding of the patient [15, 60] and adopting a collaborative approach [38]. This enables the midwife to be involved with, and recognize what especially matters for each individual patient [5, 39]. It has been stressed that understanding is created by looking at each patient in their current situation [57] and generating unique applications according to each particular set of circumstances [61].

When midwives have the ability to treat women as individuals [4], compassionate care can flow. Participants felt that there should be a stronger emphasis on an individualized approach to care, since women want to be understood by the practitioner caring for them [62]. Participants felt that an important aspect of compassion is seeing your patient (in the words of one participant) as an “individual and not a duty”, which is supported by authors who highlight the importance of humanizing the patient [34, 57]. Linkage between advocacy and compassion does not appear to have been discussed in the literature and is thus considered to be a unique finding in this study.

The issue of empathy came up frequently in participants’ descriptions of compassion, and is supported in the literature [2–4, 7, 10, 62]. One study linked compassion with trustworthiness and patience [33], and this also came through as important characteristics of compassion in this study. Davison et al. [2015] noted that labouring woman prefer midwives who give individualized attention and accommodate the particular needs of each childbearing woman; reference is also made by the authors to reaching a shared goal [63]. Berg et al. [1996] stress the importance of supporting and guiding the labouring woman according to her own terms so that she has a sense of control [64].

Once the midwife has connected with and understood the woman, she can act in a way that matters to the labouring woman and execute the care [58]. Compassion is frequently defined as an action-oriented state [5, 39] in which activities are carried out [7, 28, 61] and the deeds-based character of compassion [35] is shown by small acts of kindness [5, 39, 57] carried out in an attempt to alleviate discomfort [31, 37, 56]. This corresponds with descriptions participants gave of emotional, instrumental and informational care and support. Compassion shown through presence of the midwife, as described by participants, is also reported in the literature [3, 4, 7, 10, 60].

Characteristics of compassion found in long-term care were attentiveness, involvement and helping, and were found to be motivational in achieving agreed-upon goals [60], also emerged in the theme *action through care and support*. Midwives determination to support and encourage women reflects the affective nature of compassion [7].

A paucity of descriptions of compassion in midwifery care appeared to be a limitation in the early stages of the study but as the study progressed this became a strength, since there is a definite gap in the literature regarding midwives’ perception of compassion. This empirical study is thus able to contribute to the body of knowledge, as it identified unique findings as well as providing a starting point for continued future investigations into the concept.

A limitation of the study was that only midwives with an active Facebook account were eligible for inclusion in the study, which is not representative of the general midwifery population, as not all midwives have access to the internet or Facebook accounts [44]. This limitation is acknowledged, although representativeness is not pivotal in this qualitative study [48]. The sample obtained in this study represents a diverse selection of midwives from multiple countries, allowing multiple viewpoints to be analysed and considered, which is a strength in a qualitative study [40]. However, it would have been beneficial to have asked participating midwives in which country they reside, in order to describe the sample better.

## Conclusion

Overall, this study reveals that midwives view compassionate care as a process of connecting with the patient so that they develop an individual understanding of the patient shown through action focused on care and support. Characteristics that are considered of prime importance was displaying good interpersonal skills, conduct based on dignity and respect, establishing trust, showing empathy, prioritising maternal choice, providing emotional support, assistance through instrumental care and continuous informational support. It is recommended that further exploration be undertaken to develop an accepted conceptual definition of compassionate care in midwifery. The description of compassionate care as provided in this study could be used as an important starting point for future research investigating the concept in midwifery.

## Data Availability

The datasets used and/or analysed during the current study are available from the corresponding author on reasonable request.

## Abbreviations

ICM: International Confederation of Midwives
NWU: North-West University
SNS: Social Networking Site
WHO: World Health Organization
